# Injury-Dependent Retention of Intraportally Administered Mesenchymal Stromal Cells Following Partial Hepatectomy of Steatotic Liver Does Not Lead to Improved Liver Recovery

**DOI:** 10.1371/journal.pone.0069092

**Published:** 2013-07-18

**Authors:** Nele Boeykens, Peter Ponsaerts, Annemie Van der Linden, Zwi Berneman, Dirk Ysebaert, Kathleen De Greef

**Affiliations:** 1 Laboratory of Experimental Surgery, Antwerp Surgical Training and Research Centre, University of Antwerp/University Hospital of Antwerp, Antwerp, Belgium; 2 Laboratory of Experimental Hematology, Vaccine and Infectious Disease Institute (Vaxinfectio), University of Antwerp, Antwerp, Belgium; 3 BioImaging Laboratory, University of Antwerp, Antwerp, Belgium; University of Navarra School of Medicine and Center for Applied Medical Research (CIMA), Spain

## Abstract

The aim of this study was to evaluate the effect of bone marrow-derived mesenchymal stromal cell (BM-MSC) administration on liver function following partial hepatectomy (PHx) of methionine/choline-deficient (MCD) diet induced steatotic livers in rodents. Here we identified and validated serum cholinesterase (CHE) and triglyceride (TG) levels as non-invasive markers to longitudinally monitor rat liver function. Using *in vivo* bioluminescence imaging, retention of BM-MSC in the liver was observed following intraportal administration, but not after intravenous administration. Therefore, BM-MSC were intraportally delivered to investigate the effect on liver recovery and/or regeneration after PHx. However, despite recovery to normal body weight, liver weight and NAS score, both serum CHE and TG levels of non-treated and cell-treated rats with PHx after MCD diet remained significantly lower as compared to those of control rats. Importantly, serum CHE levels, but not TG levels, of cell-treated rats remained significantly lower as compared to those of non-treated rats, thereby warranting that certain caution should be considered for future clinical application of IP BM-MSC administration in order to promote liver regeneration and/or function.

## Introduction

Although regeneration of healthy liver tissue following major partial hepatectomy (PHx) is a well-orchestrated phenomenon carried out by different mature liver cell types [1], currently little is known regarding liver regeneration following PHx under pathological conditions. Worldwide, steatosis is the most common chronic liver disease, with a prevalence of 10-20% in lean population and 50-75% in obese population [2]. Hepatic steatosis can progress to non-alcoholic steatohepatitis (NASH), cirrhosis and development of hepatocellular carcinoma. In this context, experimental studies inducing steatosis in animal models and subsequent monitoring of liver regeneration, have already reported impaired liver regeneration [3,4]. Moreover, current clinical observations suggest an increased risk of performing PHx in patients in the presence of severe steatosis [5–7]. Therefore, (stem) cell-based therapeutic intervention gained increasing interest to overcome the limited regenerative potential of diseased liver tissue following PHx.

Despite hepatocyte transplantation being investigated as an alternative strategy for liver transplantation in metabolic, chronic and even acute liver failure [8], the limited availability and poor proliferative potential of freshly isolated (or *in vitro* cultured/differentiated) adult human hepatocytes hinders its current application [9–11]. Alternatively, in line with the current development of stem cell-based therapeutics for tissue and organ repair, mesenchymal stromal cells (MSC) are suggested to exert significant beneficial effects on the regeneration of injured liver tissue [12], although it is not clear whether the beneficial effect of MSC grafting is direct or indirect. While some studies suggest parenchymal penetration, functional engraftment or hepatocyte differentiation of MSC after transplantation in damaged liver tissue [13,14], others attribute their beneficial influence to the upregulation of hepatic regeneration-associated genes or paracrine effects on stimulation of endogenous regeneration within the injured liver [15–17].

Based on current pre-clinical evidence for MSC treatment to improve liver regeneration, in this study we aimed to investigate the potential clinical benefit of autologous bone marrow derived (BM)-MSC administration on liver regeneration following PHx after a 4 week methionine/choline-deficient (MCD) diet, thereby simulating clinical manifestation of steatosis in rodents [2]. For this, we first evaluated multiple longitudinal evaluation parameters under various conditions of MCD diet and/or PHx, and validated several blood serum parameters to be useful for this purpose. After selection of a suitable MCD + PHx model for studying long term liver regeneration, we next evaluated the *in vivo* biodistribution of BM-MSC using *in vivo* bioluminescence imaging (BLI) following intravenous or intraportal cell administration. Finally, our data show the long-term clinical evolution of liver regeneration in the MCD + PHx model following intraportal BM-MSC administration one week after PHx.

## Materials and Methods

### Animals

Female wild type Lewis rats (weighing 130-180 g, n=110) were obtained via Charles River Laboratories (strain code 004). Rats were kept in normal day-night cycle (12/12) with access to food and water *ad libitum*. Rats were subjected to control diet or methionine/choline-deficient (MCD) diet (Harlan Laboratories, Inc.) *ad libitum*. All experimental procedures were approved by the Ethical Committee for Animal Experiments of the University of Antwerp (approval no. 2008/19).

### Partial hepatectomy (PHx)

PHx (70%) of rat was performed as previously described by Mitchell and Willenbring [18], with minor modifications. Briefly, general gas anaesthesia was induced and maintained by a mixture of O_2_ and N_2_O (0.5 l/min and 1.5 l/min) and isoflurane (Isoflo®, 4% for induction and 2% for maintenance). All interventions were performed under sterile conditions, while body temperature was kept on 37°C using a heating pad with feedback control by an intrarectal placed sensor. For PHx, the abdomen was opened via a midline skin and muscle incision. The median and left lateral lobe were ligated and resected. The resected liver specimen was immediately weighed in order to estimate liver mass. After PHx, 1 ml 0,9% NaCl was given intraperitoneally and the abdomen was closed with a silk running suture. Next, rats were allowed to recover from anaesthesia under heat-producing lamps.

### Peripheral blood serum analysis

Peripheral blood was taken weekly via the tail vein (600 μl) and collected in 600 μl multivette tubes (Sarstedt). Following blood clotting (30 min.), samples were centrifuged at 1500 g for 10 minutes. Next, serum was collected and stored at -20°C until analysis. Serum cholinesterase (CHE), total serum bilirubin (TSB) and serum triglycerides (TG) were determined in duplicate by the central laboratory of the Antwerp University Hospital (UZA) using a Dimension Vista 1500 Intelligent Lab System (Siemens).

### Bone marrow-derived mesenchymal stromal cell (BM-MSC) culture

BM-MSC cultures from wild type female Lewis rats were established as previously described [19]. Briefly, bone marrow was flushed from dissected tibia and femurs, washed twice with phosphate-buffered saline (PBS) and plated in a T75 culture flask in 20 ml ‘complete isolation medium’ (CIM), consisting of RPMI-1640 medium (Invitrogen) supplemented with 8% horse serum (HS, Invitrogen), 8% foetal calf serum (FCS, Hyclone), 100 U/ml penicillin (Invitrogen), 100 mg/ml streptomycin (Invitrogen), and 1.25 mg/ml amphotericin B (Invitrogen). For a period of two weeks, CIM was replaced every 3 to 4 days. Upon confluence, cultured cells were harvested using trypsin-EDTA (Invitrogen) treatment and replated in a new T75 culture flask in 20 ml CIM. Stromal cell outgrowth in this culture was termed passage 1 and further expanded in 'complete expansion medium' (CEM), consisting of Iscove modified Dulbecco’s medium (IMDM, Cambrex) supplemented with 8% FCS, 8% HS, 100 U/ml penicillin, 100 mg/ml streptomycin and 1.25 mg/ml amphotericin B. For routine cell culture, BM-MSC cultures were harvested twice a week using trypsin–EDTA treatment and passaged at a 1:3 ratio in 15 mL CEM in T75 culture flasks.

### Characterization of BM-MSC cultures by RT-PCR

Total RNA was isolated from BM-MSC using the AllPrep DNA/RNA mini kit (Qiagen), according to manufacturer’s instructions. Next, cDNA was synthesized from extracted total RNA using the Omniscript RT kit (Qiagen), according to manufacturer’s instructions. Subsequently, a PCR mix was prepared consisting of prepared cDNA (1 μg), buffers from the TaqPCR Core kit (Qiagen) and one of the following primer pairs (25 pmol each): Alpha-fetoprotein (AFP) (Forward: ACCTGACAGGGAAGATGGTG, Reverse: GCAGTGGTTGATACCGGAGT), Albumin (Forward: TCTGCACACTCCCAGACAAG, Reverse: AGTCACCCATCACCGTCTTC), Vimentin (Forward: ACGAGTACCGGAGACAGGTG, Reverse: TCCAGCAGCTTCCTGTAGGT), GAPDH (Forward: ACCACAGTCCATGCCATCAC, Reverse: TCCACCACCCTGTTGCTGTA) or CD45 (Forward: TGTGAACATACGGATTGTGAA, Reverse: CTATGTCTGGTGTGCAGTTTG). The PCR reaction was run on a P2X Thermal Cycler (Thermo Electron cooperation) according to the following program: denaturation at 95°C for 1 minute, annealing at 56°C for 1 minute and extension at 72°C for 1 minute for 30 cycles, with a final extension at 72°C for 10 minutes. Next PCR products were separated on 1.5% agarose gel, stained with gel red and amplified products were visualized using a standard UV light source.

### Lentiviral vector transduction of BM-MSC

BM-MSC were transduced with a lentiviral vector encoding both the eGFP and luciferase reporter proteins [20], according to previously optimized procedures [19,21–24]. Briefly, cells were overnight seeded in a 24-well plate at 10,000 cells per well in 750 μl CEM. The next day, lentiviral vector (2.45x10^5^ TU) was added for 48 hours. Next, transduced BM-MSC cultures were washed and further expanded in CEM. Before experimental use, transduced BM-MSC, further named as BM-MSC/eGFP-Luc, were passaged at least 4 times. Transgene expression was determined by flow cytometry and *in vitro* luminescence assay.

### Flow cytometry

Immunophenotyping of BM-MSC cultures derived from Lewis rats was performed using the following antibody combinations: phycoerythrin (PE)-labeled mouse anti-rat CD45 (Becton Dickinson, 554878), PE-labeled mouse anti-mouse/rat CD90.1 (eBioscience, 12-0900-81), fluorescein isothiocyanate (FITC)-labeled hamster anti-rat CD29 (Becton Dickinson, 561796), and mouse anti-rat CD73 (Becton Dickinson, 551123) in combination with FITC-labeled goat anti-mouse IgG1 secondary antibody (Jackson ImmunoResearch, 115-095-205). Before staining, harvested cells were washed twice with PBS and resuspended in PBS at a concentration of 5 × 10^5^ cells/ml. For antibody staining, 1 μg of antibody was added to 100 μl of cell suspension for 20 min at 4°C. The same procedure was applied in case of secondary antibody staining. Following incubation, cells were washed once with PBS, resuspended in 0.5 mL PBS, and analyzed using an Epics XL-MCL analytical flow cytometer (Beckman Coulter). For determination of eGFP transgene expression, harvested BM-MSC or BM-MSC/eGFP-Luc cultures were washed once with PBS, resuspended in PBS and directly analysed using an Epics XL-MCL analytical flow cytometer. For all analyses, cell viability was assessed through addition of GelRed (1× final concentration, Biotum) to the cell suspension immediately before flow cytometric analysis. At least 10,000 cells per sample were analysed and flow cytometry data were analysed using FlowJo software.

### In vitro luminescence assay

Luciferase activity in cultured BM-MSC and BM-MSC/eGFP-Luc (1 x 10^5^ cells per assay) was measured using the commercial Bright-Glo luciferase assay system (Promega), according to the manufacturer’s instructions. The luminescence signal was quantified using a microplate reader (Tecan, Germany) and expressed in relative light units (RLU).

### Cell administration

Following harvesting of BM-MSC or BM-MSC/eGFP-Luc cell populations via trypsin/EDTA treatment, cells were washed twice with PBS and resuspended at a concentration of 3,33 × 10^6^ cells/mL in PBS. Cell preparations (mean viability > 95%) were kept on ice until administration. For, intravenous (IV) cell administration, 150 μl (0.5 x 10^6^ cells) was injected via the tail vein, using an insulin syringe (29G) (Terumo). For intraportal (IP) cell administration, the peritoneal cavity was opened under general anaesthesia (as described above) in order to expose the portal vein. Following puncture of the vein using an insulin syringe (29G), 150 μl of cell suspension (0.5 x 10^6^ cells) was slowly injected. Next, the needle was retracted and bleeding was stopped using an absorbable gelatine sponge. Finally, the abdomen was closed with a silk running suture and rats were allowed to recover from anaesthesia under heat-producing lamps.

### In vivo bioluminescence imaging (BLI)

In order to determine *in vivo* biodistribution of IV and IP administered BM-MSC/eGFP-Luc, whole body BLI was performed as previously described [25]. Briefly, at 2h and 24h following cell administration, rats were anaesthetized using a mixture of O_2_ (0.5 l/min) and isoflurane (Isoflo®, 4% for induction and 2% for maintenance), followed by an intravenous injection of D-luciferin (100 mg/kg body weight dissolved in PBS; Promega, Benelux) via the tail vein. Immediately after D-luciferin administration, rats were imaged for 5 min using a real-time Photon-imager system (Biospace Lab). At the end of every acquisition, a photographic image of the animal was obtained to which the bioluminescence image was superimposed by the M3Vision analysis software (Biospace Lab). The most intense bioluminescence signal is represented in red while the weakest signal is shown in blue. Signal intensities on the scale bars are given in photons/s/sr/cm^2^ from a 5-min time period. Additionally, selected animals were sacrificed directly after whole body BLI in order to remove organs (lung, kidney, spleen, heart and liver) for *ex vivo* BLI analysis.

### Histological analysis

After PHx, the resected liver parts are directly weighed. At the end of the study, the livers were removed and also directly weighed in order to calculate liver regeneration, in weight (see below). Next, resected liver part and remnant liver lobe (right lateral lobe) were divided in two and fixed in both methacarn and formol/calcium. Following fixation in methacarn (60% methanol, 10% acetic acid, 30% trichlorethane) for 4h at room temperature liver tissue was rinsed in 70% ethanol. Methacarn-fixed tissue samples were then embedded in low-melting point paraffin for preparation of 5 μm sections. Following fixation in formol/calcium (4% formaldehyde in 0.1M Na-cacodylate pH7.4, 1% Ca-chloride) for 1.5h at room temperature, liver tissue was rinsed in wash buffer (0.1M Na-cacodylate pH7.4 in water, 1% Ca-chloride). Formol/calcium-fixed tissue samples then frozen in liquid nitrogen for preparation of 6 μm cryosections. Standard H&E (Haemaluin Carazzi & Eosine) staining was performed on paraffin embedded sections and used to visualize general liver morphology and to determine the degree of hepatocyte ballooning. Standard Oil Red O staining was performed on cryosections and used to determine the degree of steatosis. Immunohistochemical staining for OX-1 was performed in combination with PAS (periodic acid and Schiff reagent)-staining on paraffin embedded sections and used to determine the degree of lobular inflammation. All stainings were performed as described previously [26] and evaluation of liver tissue was done according to the standard NAS-scoring method [27] by a blind investigator. Hepatocyte ballooning was evaluated in 10 high power fields (HPF) at magnification x40, whereas steatosis and lobular inflammation were evaluated in 10 HPF at magnification x20.

### Calculation of liver weight

In the first experimental set-up (i.e. characterization of the MCD/PHx model), whole liver weight of rats after 4 weeks of CONTROL diet (group A*) or after 4 weeks of MCD diet (group C*) was determined on a separate animal group and used as a mean starting point to evaluate liver injury and/or regeneration after PHx in the experimental groups. In the second experimental setup (i.e. cell therapy in MCD/PHx model), whole liver weight of rats after 4 weeks of CONTROL diet or after 4 weeks of MCD diet was estimated based on the above calculated body weight / liver weight ratios for CONTROL and MCD-treated rats. Further estimation of liver weight directly after PHx was calculated based on the actual weight of the resected liver parts. Evaluation of liver weight at the end of the study was done based on the actual weight of the dissected livers.

### Statistical analysis

A linear mixed effects model was fitted to model the evolution of body weight, serum CHE, TSB and serum TG over time and to study the effect of MCD and/or PHx on this evolution. The fixed part of the model contains an intercept, time-effect, MCD-effect, prior-MCD-effect, PHx-effect and all two- and three-way interactions between them. The repeated measures character of the data is taken into account by including a subject-specific intercept and random slope in the random part of the model. For liver weight, a repeated measures ANOVA with group as between- and time as within subjects factor, is used to compare the 6 groups at the 3 time-points. To adjust for multiple testing, false discovery rate correction (FDR) is applied. For NAS scoring, Mann-Whitney U Test was used to compare scores (median (Q1-Q3)) between groups. All analyses were done in SAS 9.2 or IBM SPSS Statistics 20.

## Results

### Recovery assessment following methionine/choline-deficient (MCD) diet and/or partial hepatectomy (PHx) in rat

**Figure 1:**
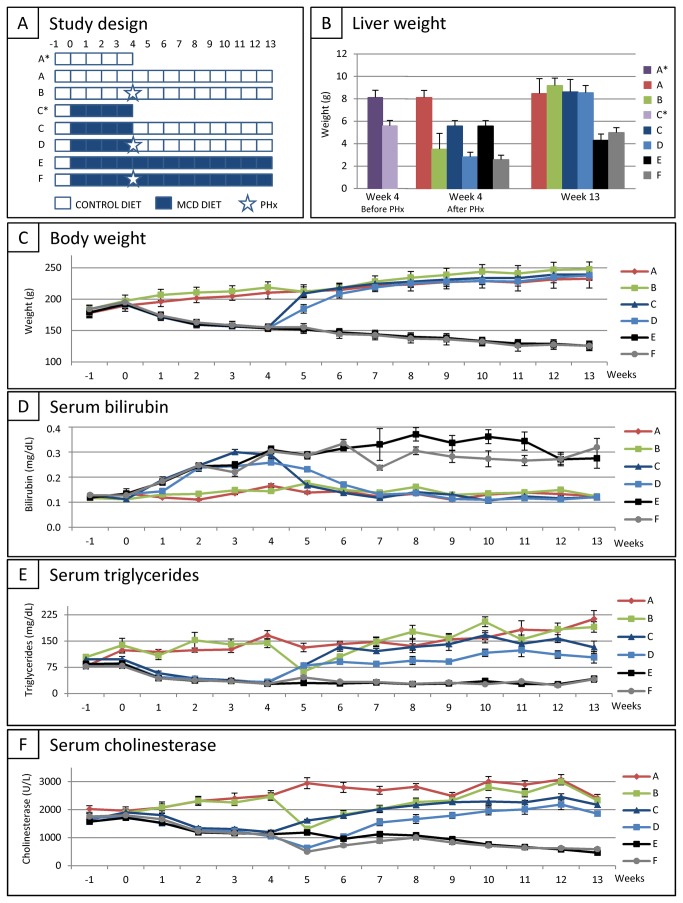
Recovery assessment following partial hepatectomy (PHx) and/or methionine/choline-deficient (MCD) diet in rat. (A) Study design. (B) Mean liver weight for each experimental group at week 4 before PHx (left panel), at week 4 directly after PHx (middle panel) and at week 13 (end of study, right panel). (C) Evolution of body weight for each experimental group. Data are expressed in gram ± standard error. (D) Evolution of total serum bilirubin (TSB) level for each experimental group. Data are expressed in milligram per decilitre ± standard error. (E) Evolution of serum triglyceride (TG) level for each experimental group. Data are expressed in milligram per decilitre ± standard error. (F) Evolution of serum cholinesterase (CHE) level for each experimental group. Data are expressed in units per litre ± standard error. For each experimental group at each time point n=10.

Animal recovery following MCD diet and/or PHx was evaluated based on: (i) liver weight before PHx, after PHx and at the end of the study, (ii) longitudinal measurement of body weight, (iii) longitudinal measurement of multiple serum parameters, and (iv) liver NAS scoring at the end of the study. To select the most ideal experimental set-up for further cell therapy studies, female wild type Lewis rats were divided into six groups (Figure 1A): (A) control rats fed with control diet for 13 weeks (CONTROL DIET, *n=10*), (B) rats fed with control diet for 13 weeks and subjected to PHx at week 4 (CONTROL DIET + PHx, *n=10*), (C) rats fed with MCD diet for 4 weeks followed by control diet for 9 weeks (MCD DIET + CONTROL DIET, *n=10*), (D) rats fed with MCD diet for 4 weeks, subsequently subjected to PHx, followed by control diet for 9 weeks (MCD DIET + PHx + CONTROL DIET, *n=10*), (E) rats fed with MCD diet for 13 weeks (MCD DIET, *n=10*), and (F) rats fed with MCD diet for 13 weeks and subjected to PHx at week 4 (MCD DIET + PHx, *n=10*).

For determination of liver weight after 4 weeks of control diet or MCD diet, 8 additional rats were divided into 2 groups (Figure 1A): (A*) control rats fed with control diet for 4 weeks (CONTROL DIET, *n=4*) and (C*) rats fed with MCD diet for 4 weeks (MCD DIET, *n=4*). As shown in Figure 1B (left panel), a significant decrease in liver weight is observed after 4 weeks of MCD diet (GROUP A* vs. C*; 8,13 g ± 0,63 g vs. 5,59 g ± 0,48 g; p<0.0001). In order to calculate whole liver weight after PHx at week 4, all resected liver parts of control diet rats (GROUP B) or MCD diet rats (GROUP D and F) were weighed and subtracted from the obtained control values (GROUP A* and C*). Based on these data, Figure 1B (middle panel) shows the estimated whole liver weight for each experimental group at week 4 of the experimental set-up. Finally, at the end of the experiment (week 13), liver weight of all rats used in this study was determined and is shown in Figure 1B (right panel). Liver weight returned to control levels (GROUP A) after CONTROL DIET + PHx (GROUP B), MCD DIET (GROUP C) and MCD DIET + PHx (GROUP D) following 9 weeks of CONTROL DIET. In contrast, liver weight remained significantly lower as compared to liver weight of control rats following continuation of the MCD diet for 9 weeks (GROUP A vs. E and F; 8,48 g ± 1,32 g vs. respectively 4,31 g ± 0,56 g and 4,99 g ± 0,45 g; for both p<0.0001).

A significant decrease in total body weight is observed after 4 weeks of MCD diet (Figure 1C) (GROUP A-B vs. C–F; 214,7 g ± 10,14 g vs. 154,43 g ± 4,63 g; p<0.0001). Upon cessation of MCD diet (GROUP C and D), body weight immediately increased to control levels. In contrast, body weight remained significantly lower following continuation of the MCD diet for 9 weeks (GROUP A vs. E and F; 232,9 g ± 10,14 g vs. respectively 125,8 g ± 6,32 g and 125,75 g ± 7,74 g; for both p<0.0001).

A significant increase in total serum bilirubin (TSB) levels is observed after 4 weeks of MCD diet (Figure 1D) (GROUP A-B vs. C–F; 0,155mg/dL ± 0,024 mg/dL vs. 0,290 mg/dL ± 0,044 mg/dL; p<0.0001). Whereas TSB levels remained increased following continuation of the MCD diet for 9 weeks (GROUP A vs. E and F; 0,123 mg/dL ± 0,015 mg/dL vs. respectively 0,275 mg/dL ± 0,127 mg/dL and 0,319 mg/dL ± 0,101 mg/dL; for both p<0.0001), TSB levels immediately decreased to control levels upon cessation of the diet (GROUP C and D).

As shown in Figure 1E, a significant decrease in triglyceride (TG) levels is observed after 4 weeks of MCD diet (GROUP A-B vs. C–F; 155,5 mg/dL ± 41,2 mg/dL vs. 30,1 mg/dL ± 6,2 mg/dL; p<0.0001) and after PHx alone (GROUP A vs. B; 131,7 mg/dL ± 39,1 mg/dL vs. 63,0 mg/dL ± 9,2 mg/dL; p<0.0001). Unlike TG levels of hepatectomized control rats (GROUP B) and non-hepatectomized MCD diet-fed rats (GROUP C), which recover to control levels within the study period, TG levels of MCD diet-fed animals subjected to PHx (group D) did not equal those of control rats at 9 weeks after PHx (GROUP A vs. D; 213,3 mg/dL ± 75,0 mg/dL vs. 103,4 mg/dL ± 49,3 mg/dL; p=0.0125). Following continuation of the MCD diet for 9 weeks, serum TG levels remained significantly lower as compared to serum TG levels of control rats (GROUP A vs. E and F; 213,3 mg/dL ± 75,0 mg/dL vs. respectively 42,2 mg/dL ± 24,2 mg/dL and 41,1 mg/dL ± 25,4 mg/dL; for both p<0.0001).

In addition, serum cholinesterase (CHE) levels were affected both by MCD diet and PHx. As shown in Figure 1F, while MCD diet already significantly decreased serum CHE levels after 4 weeks (GROUP A-B vs. C–F; 2491U/L ± 467 U/L vs. 1122 U/L ± 161 U/L; p<0.0001), a respectively initial decrease (in rats with CONTROL DIET, GROUP B) and further decrease (in rats with MCD DIET, GROUP D and F) of serum CHE levels was observed during the first week after PHx (CONTROL DIET + PHx; 1315 U/L ± 323 U/L; GROUP B at week 5 vs. GROUP A-B at week 4: p<0.0001; MCD DIET + PHx; 568 U/L ± 138 U/L; (GROUP D+F at week 5 vs. GROUP C–F at week 4: p<0.0001). Serum CHE levels recovered to control levels after PHx in rats with CONTROL DIET (GROUP B) and after cessation of the MCD diet in non-hepatectomized rats (GROUP C). In contrast, serum CHE levels of MCD diet-fed animals subjected to PHx (GROUP D) displayed increasing CHE levels but did not equal those of control rats at the end of the experiment (GROUP A vs. D, 2418 U/L ± 412 U/L vs. 1860 U/L ± 306 U/L; p=0.0125). Following continuation of the MCD diet for 9 weeks, serum CHE levels remained significantly lower as compared to serum CHE levels of control rats (GROUP A vs. E and F; 2418 U/L ± 412 U/L vs. respectively 464 U/L ± 115 U/L and 592 U/L ± 140 U/L; for both p<0.0001).

**Figure 2:**
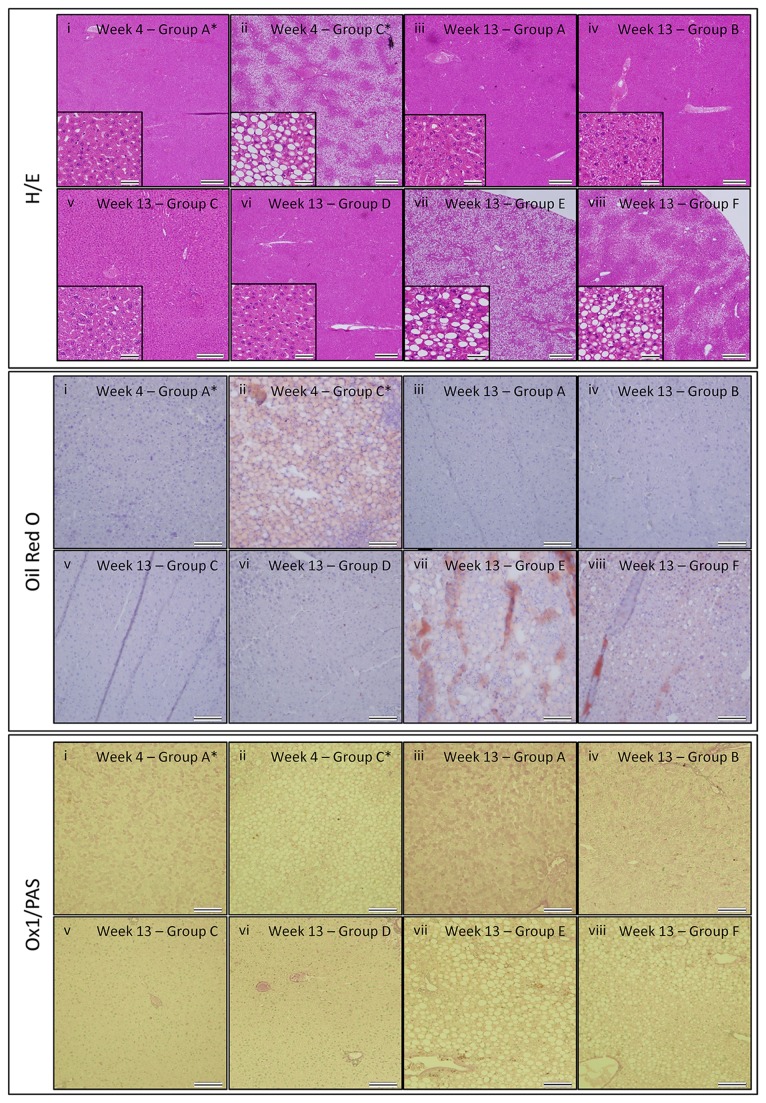
Histological analysis of liver tissue following PHx and/or MCD. Haematoxylin-eosin (H–E) staining (upper panel), Oil Red O staining (middle panel) and PAS/OX-1 staining (lower panel). (i) after 4 weeks of control diet (GROUP A*), (ii) after 4 weeks of MCD diet (GROUP C*), (iii) after 13 weeks of control diet (GROUP A), (iv) after 13 weeks of control diet with PHx at week 4 (GROUP B), (v) after 4 weeks of MCD diet followed by 9 weeks of control diet (GROUP C), (vi) after 4 weeks of MCD diet followed by PHx and 9 weeks of control diet (GROUP D), (vii) after 13 weeks of MCD diet (GROUP E), (viii) after 13 weeks of MCD diet with PHx at week 4 (GROUP F). Representative images were chosen out of 3 mice analysed per experimental group. For H-E staining, scale bars in the main image indicate 500 μm, while scale bars in the inset images indicate 50 μm. For Oil Red O and PAS/OX-1 staining, scale bars indicate 100 μm.

At the time point of PHx and at the end of the study, liver tissue was prepared for histological analyses. Following H&E, Oil Red O and OX1/PAS staining, histological evaluation of tissue sections was performed according to the NAS-scoring method, based on the degree of hepatocyte ballooning, steatosis, and lobular inflammation (for GROUP A-D and E-F, 3 and 5 livers per group were analysed, respectively). As shown by the representative images in Figure 2 and the detailed NAS scoring in table 1, livers display significant steatosis and hepatocyte ballooning following 4 weeks of MCD diet (Figure 2B, GROUP D+F, median NAS score 3 (Q1-Q3: 3–3.125)) as compared to livers from control rats (Figure 2A, GROUP B, NAS score 0), indicating successful induction of steatosis by the MCD diet. Of note, while no lobular inflammation was observed after 4 weeks of MCD diet, portal inflammation was clearly present (although the latter is not included in the NAS scoring method). Further, independent of preceding MCD diet and/or PHx (GROUPS B–D), after 9 weeks on CONTROL diet liver tissue regenerated without significant signs of steatosis, lobular inflammation or hepatocyte ballooning (Figure 2D–F, GROUP B–D, for all NAS score 0), as compared to livers from control rats of the same age (Figure 2C, GROUP A, NAS score 0). However, following long-term administration of MCD diet (13 weeks), steatosis, portal inflammation (however, not included in NAS score) and hepatocyte ballooning remains (Figure 2G, GROUP E, median NAS score 4 (Q1-Q3: 3.5–4)), which did not worsen as compared to 4 weeks of MCD diet (see above). Notably, rats continuously receiving MCD diet for 13 weeks with a PHx at week 4, displayed a reduction in liver steatosis (Figure 2H, GROUP F, median NAS score 2.5 (Q1-Q3: 2-3)) as compared to rats under MCD diet without PHx (GROUP E vs. F, p=0.032).

**Table 1:** Calculation of the median NAS score for the different experimental groups.

**Group**	**Week**	**Median score (Q1-Q3) Ballooning**	**Median score (Q1-Q3) Steatosis**	**Median score (Q1-Q3) Lobular inflammation**	**Median score (Q1-Q3) NAS**
B	4	0	0	0	0
D+F	4	0 (0 - 0.125)	3 (3 - 3)	0	3 (3-3.125)
A	13	0	0	0	0
B	13	0	0	0	0
C	13	0	0	0	0
D	13	0	0	0	0
E	13	1 (0.5-1)	3 (3 - 3)	0	4 (3.5-4)
F	13	0.5 (0 - 0.5)	2 (2-2)	0	2.5 (2-3)

In conclusion, based on the presented data above, experimental GROUP D (i.e. MCD diet for 4 weeks + PHx + recovery for 9 weeks on CONTROL diet) was selected for further cell therapy studies. The significant longitudinal differences in serum CHE and TG levels, which after a recovery period of 9 weeks do not equal those of control rats, allows this model to study either increased liver recovery, both in time and function, following MCD diet + PHx.

### In vivo BM-MSC biodistribution analysis following intravenous or intraportal administration

**Figure 3:**
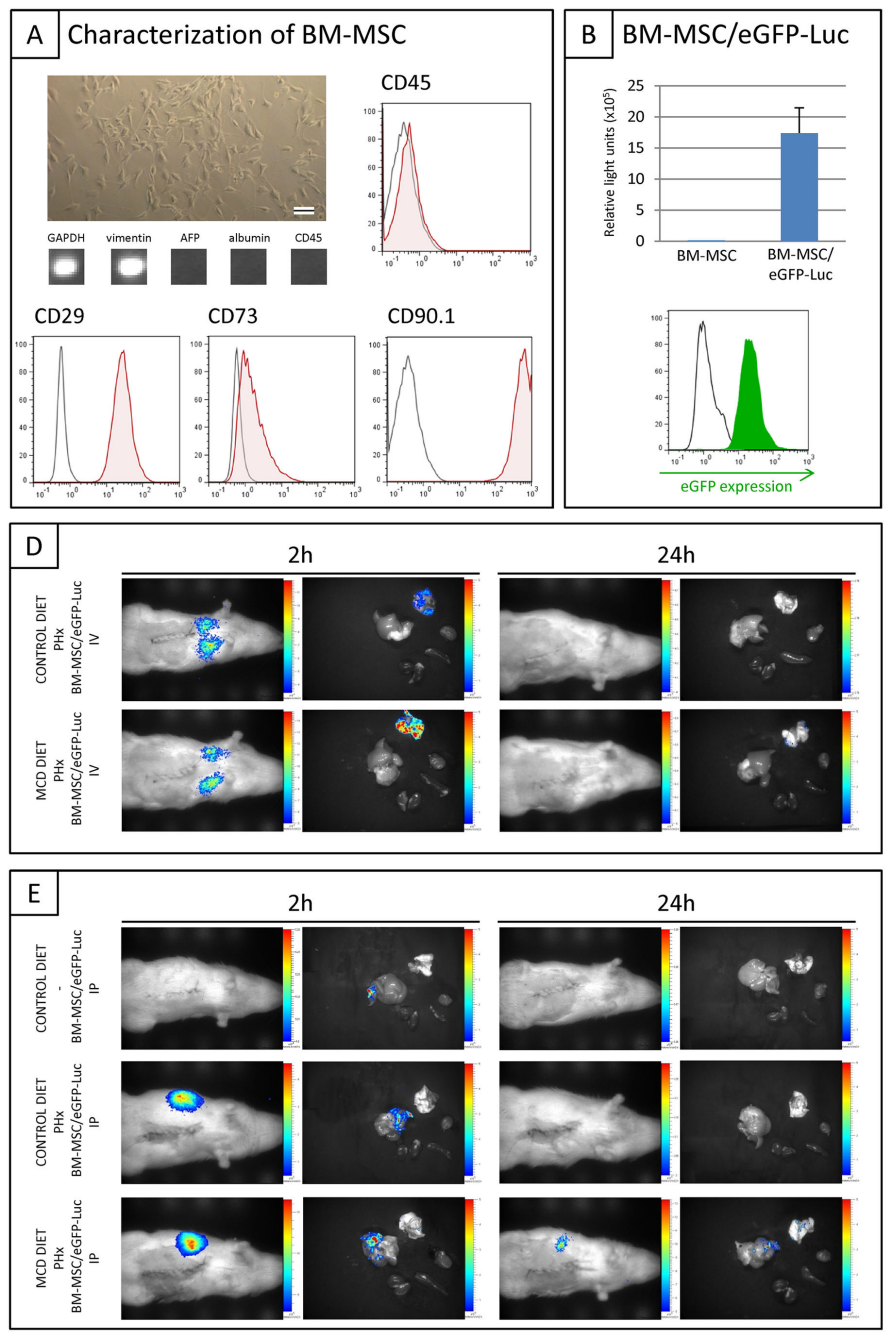
*In* *vivo* / *ex* *vivo* bioluminescence imaging (BLI) following intravenous (IV) and intraportal (IP) administration of BM-MSC/eGFP-Luc. (A) Bright field phase contrast microscopic image of cultured rat BM-MSC. The scale bar indicates 100 μm. RT-PCR analysis of cultured BM-MSC: GAPDH, vimentin, AFP, albumin, CD45. A representative output was chosen from two independent measurements at multiple passages. Flow cytometric overlay histograms showing the expression pattern of membrane proteins on BM-MSC derived from Lewis rats (i.e. expression of CD27, CD73 and CD90.1, but no expression of CD45). Open histograms: unstained control. Filled histograms: specific antibody staining. A representative histogram overlay was chosen from three independent measurements at multiple passages. (B) Overlay histogram: level of eGFP expression by control BM-MSC (open black histogram) and transduced BM-MSC/eGFP-Luc (filled green histogram). A representative histogram overlay was chosen from four independent measurements at multiple passages. Right graph: In vitro luminescence assay using 1x10^5^ control BM-MSC and transduced BM-MSC/eGFP-Luc. Data are expressed as mean relative light units ± standard error (n=4 measurements at multiple passages). (C) Representative BLI images of healthy (upper panel) and MCD diet-fed animals (lower panel) subjected to PHx at week 4 with intravenous BM-MSC/eGFP-Luc administration at week 5 (n=2). (D) Representative BLI images of control animals (upper panel) with intraportal BM-MSC/eGFP-Luc administration (n=4) and BLI images of healthy (middle panel) and MCD diet-fed animals (lower panel) subjected to PHx at week 4 with intraportal BM-MSC/eGFP-Luc administration at week 5 (n=3). Left panel: *In vivo* BLI (left picture) and *ex vivo* BLI (right picture) at 2 hours post-implantation. Right panel: *In vivo* BLI (left picture) and *ex vivo* BLI (right picture) at 24 hours post-implantation. *Ex vivo* images show the following organs in clockwise direction: lung (upper), heart, spleen, kidney, liver.

As shown in Figure 3A, BM-MSC isolated from female wild type Lewis rats are expanded under adherent culture conditions and display typical fibroblast morphology. Further RT-PCR based characterization of cultured BM-MSC showed expression of the mesenchymal-lineage marker vimentin, without apparent expression of hematopoietic (CD45) and liver-associated proteins (AFP and albumin). In addition, flow cytometric analysis indicated uniform expression of the mesenchymal markers CD29, CD90.1 and CD73, without apparent expression of the haematopoietic marker CD45. For *in vivo* cell tracking studies, cultured BM-MSC were additionally transduced with a lentiviral vector encoding both the eGFP and luciferase reporter proteins. As shown in Figure 3B, transduced BM-MSC, further named as BM-MSC/eGFP-Luc, display high level of eGFP expression (left histogram, flow cytometric analysis) and luciferase activity (right graph, *in vitro* luminescence assay). For *in vivo* cell tracking experiments, BM-MSC/eGFP-Luc were used at passage 16-18.

In order to select for an optimal administration route for BM-MSC in further therapeutic studies for liver disease, we first analysed their *in vivo* biodistribution following intravenous (IV) or intraportal (IP) administration. For IV administration, 0.5x10^6^ BM-MSC/eGFP-Luc were administered via the tail vein one week after PHx in rats receiving CONTROL diet (n=2) or MCD diet (n=2) before PHx. Using *in vivo* and *ex vivo* bioluminescence imaging (BLI), biodistribution of administered BM-MSC/eGFP-Luc was monitored at 2 and 24 hours post administration. As shown in Figure 3C, for both groups a clear *in vivo* BLI signal was detected in the lungs at 2 hours post IV cell administration, which could not be detected at 24 hours after administration. The latter was further confirmed by *ex vivo* BLI. For IP administration, 0.5x10^6^ BM-MSC/eGFP-Luc were administered via the portal vein in non-hepatectomized control rats (n=3) and one week after PHx in rats receiving CONTROL diet (n=3) or MCD diet (n=3) before PHx. As shown in Figure 3D (upper panel), no signal could be detected by *in vivo* BLI at 2 and 24 hours post administration in non-hepatectomized rats. Additional *ex vivo* BLI was able to detect a weak BLI signal in liver at 2 hours post administration, which was absent at 24 hours post administration. In contrast, *in vivo* BLI demonstrates that cell administration one week after PHx in rats receiving CONTROL or MCD diet (Figure 3D, middle and lower panel) results in the detection of a clear *in vivo* BLI signal within the liver at 2 hours post administration, which was dramatically decreased (MCD diet group, lower panel) or absent (CONTROL diet group, middle panel) at 24 hours post administration. The latter was further confirmed by *ex vivo* BLI.

In conclusion, based on the presented data above, IP cell administration was superior to IV cell administration in order to direct (via active homing or passive retention) grafted cells to the injured/regenerating liver.

### Recovery assessment following IP administration of BM-MSC after MCD diet and PHx

**Figure 4:**
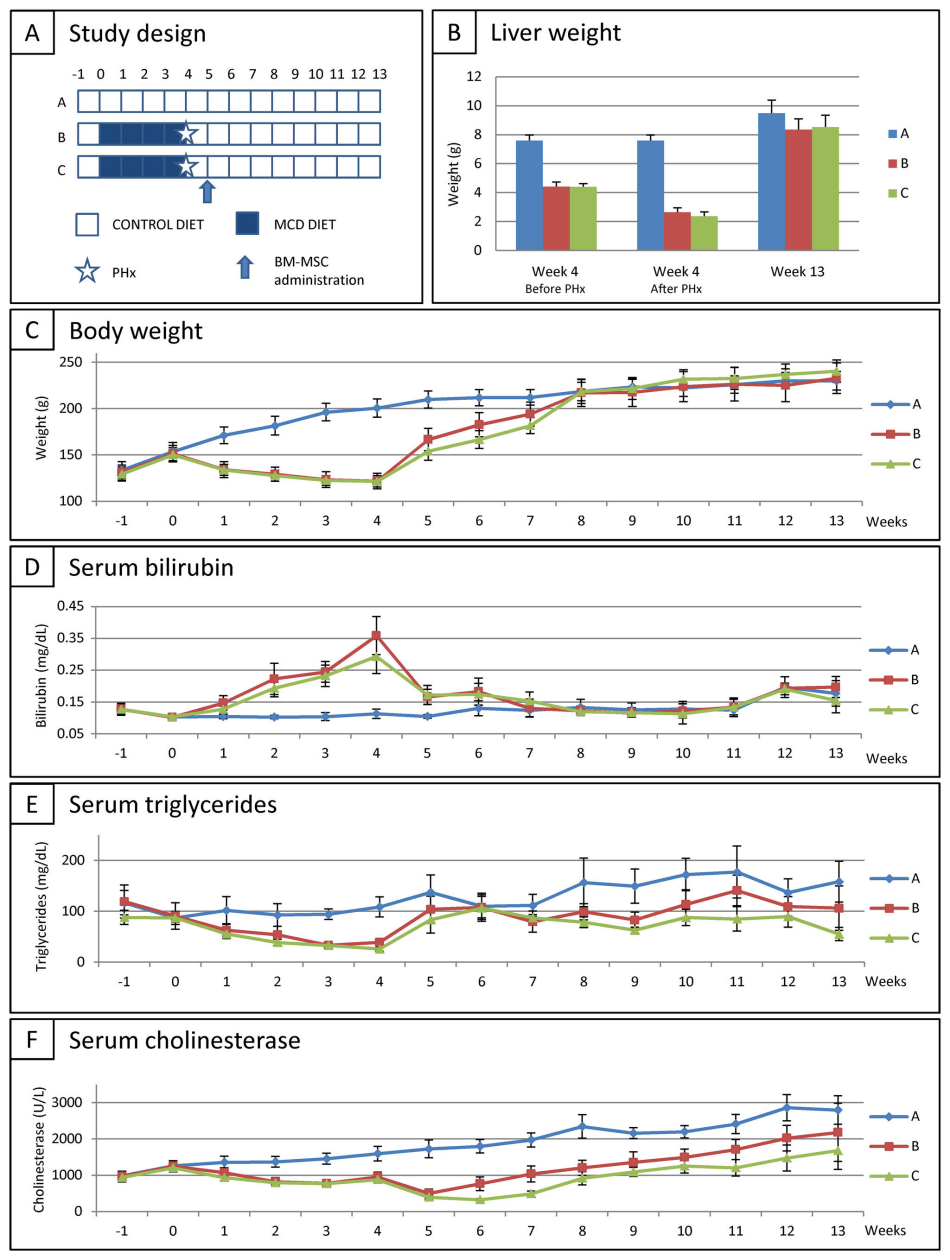
Recovery assessment following intraportal administration of BM-MSC after MCD diet and PHx in rat. (A) Study design. (B) Mean liver weight for each experimental group at week 4 before PHx (left panel), at week 4 directly after PHx (middle panel) and at week 13 (end of study, right panel). (C) Evolution of body weight for each experimental group. Data are expressed in in gram ± standard error. (D) Evolution of total serum bilirubin (TSB) level for each experimental group. Data are expressed in milligram per decilitre ± standard error. (E) Evolution of serum triglyceride (TG) level for each experimental group. Data are expressed in milligram per decilitre ± standard error. (F) Evolution of serum cholinesterase (CHE) level for each experimental group. Data are expressed in units per litre ± standard error. For each experimental group at each time point n=10.

For this experimental set-up, female wild type Lewis rats were divided into 3 groups (Figure 4A): (A) rats fed with control diet for 13 weeks (CONTROL DIET, *n=10*), (B) rats fed with MCD diet for 4 weeks, subsequently subjected to PHx at week 4, followed by control diet for 9 weeks (MCD DIET + PHx + CONTROL DIET, *n=10*), (C) rats fed with MCD DIET for 4 weeks, subsequently subjected to PHx at week 4, followed by control diet for 9 weeks, with IP BM-MSC administration (0.5x10^6^ cells) one week after PHx (MCD DIET + PHx + BM-MSC + CONTROL DIET, *n=10*). For therapeutic intervention, non-transduced parental BM-MSC were used (at passage 4) in order to avoid potential immune responses against the eGFP and Luciferase reporter proteins. Animal recovery was subsequently monitored as described in the first results section.

Although at study end liver weight of non-treated and cell-treated rats remained slightly lower as compared to liver weight of control rats (GROUP A vs. GROUP B and C; 9,49 g ± 0,90 g vs. respectively 8,35 g ± 0,74 g (p=0.0018) and 8,53 g ± 0,81 g (p=0.0178)), no significant difference was observed between liver weight of non-treated and cell-treated rats (GROUP B vs. GROUP C) (Figure 4B).

As shown in Figure 4C, 4D and 4E, no significant differences in body weight, TSB levels and serum TG levels were observed between non-treated (GROUP B) and cell-treated (GROUP C) rats, and serum TG levels of both cell-treated and non-treated rats remained significantly lower as compared to control levels (GROUP A vs. GROUP B and C, 158,3 mg/dL ± 40,1 mg/dL vs. respectively 104,6 mg/dL ± 45,9 mg/dL (p=0,0081) and 55,0 mg/dL ± 13,0 mg/dL (p=0,0001)). Additionally, as shown in Figure 4F, cell-treated rats (GROUP C) display a delay of 2 weeks before serum CHE levels start increasing as compared to non-treated rats (GROUP B). Moreover, during the whole recovery period serum CHE levels of cell-treated rats remained significantly lower as compared to non-treated rats (GROUP B vs. GROUP C, 2181U/L ± 801U/L vs. 1680 U/L ± 519 U/L; p<0.0001) and as compared to control rats (GROUP A vs. GROUP C; 2797 U/L ± 392 U/L vs. 1680 U/L ± 519 U/L; p<0.0001).

**Figure 5:**
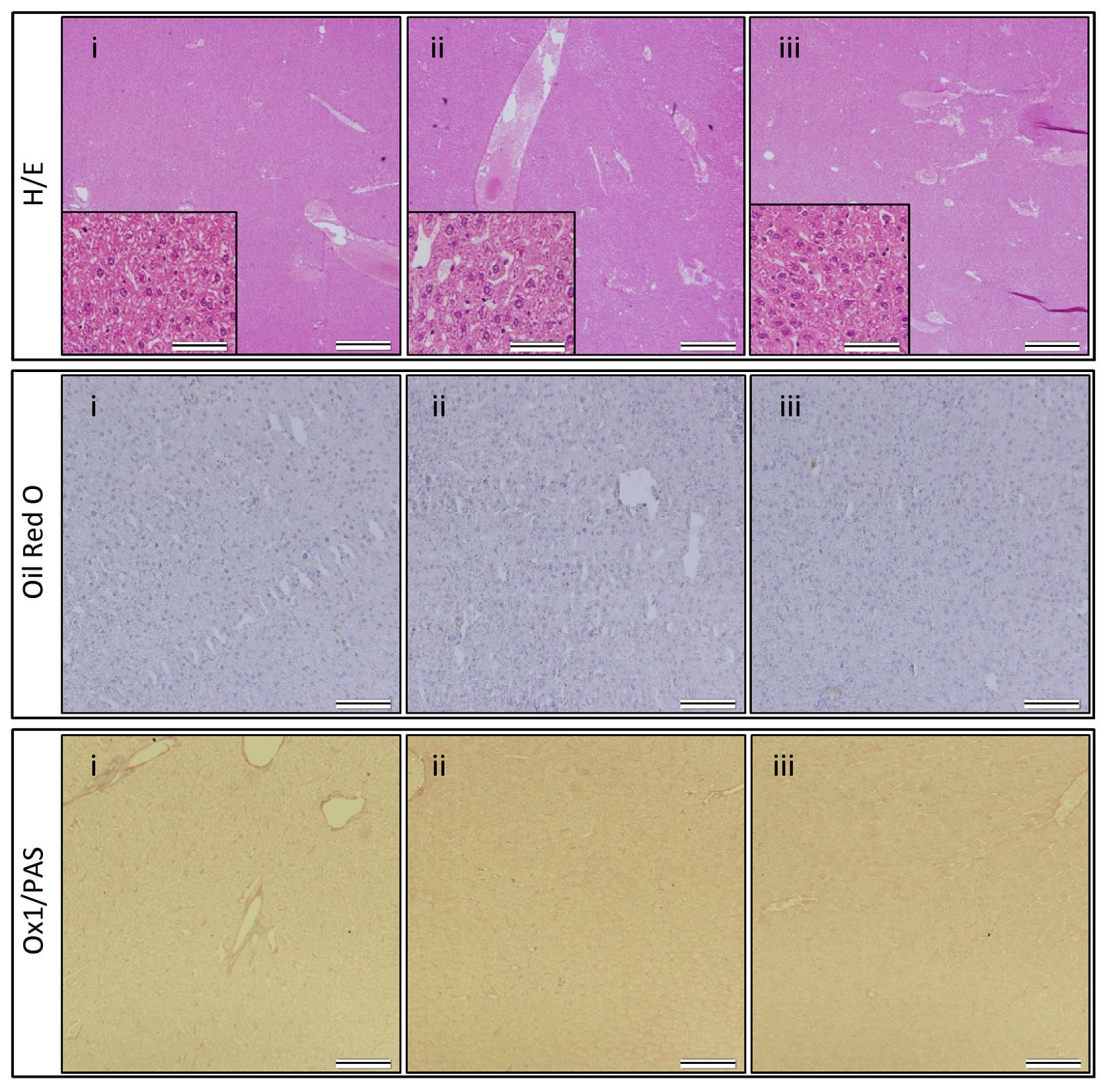
Histological analysis of liver tissue following intraportal administration of BM-MSC after MCD diet and PHx in rat. Haematoxylin-eosin (H–E) staining (upper panel), Oil Red O staining (middle panel) and PAS/OX-1 staining (lower panel). (i) after 13 weeks of control diet (GROUP A), (ii) after 4 weeks of MCD diet, followed by PHx and control diet for 9 weeks (GROUP B), (iii) after 4 weeks of MCD diet, followed by PHx, BM-MSC administration and control diet for 9 weeks (GROUP C). Representative images were chosen out of 3 mice analysed per experimental group. For H-E staining, scale bars in the main image indicate 500 μm, while scale bars in the inset images indicate 50 μm. For Oil Red O and PAS/OX-1 staining, scale bars indicate 100 μm.

Finally, at the end of the study, liver tissue was prepared for histological analyses. As shown by the representative H&E, Oil Red O, and OX1/PAS stainings in Figure 5, no significant hepatocyte ballooning, steatosis or lobular inflammation was observed in all three groups (Figure 5A–C, GROUP A-C, for all NAS score 0).

In conclusion, based on the data presented above, we cannot attribute any functional benefit to IP BM-MSC administration following PHx of steatotic livers.

## Discussion

The present study aimed to optimize a rodent model of PHx of MCD diet-induced steatotic liver, including the validation of longitudinal serum parameter measurements as a tool to monitor liver function/recovery. As shown in Figures 1 and 2, this experimental set-up is based on a 4 week MCD diet resulting in decreased total body and liver weight, decreased serum TG and CHE levels, and detectable macrovesicular steatosis and ballooning. Based on our preceding optimization experiments, a 4 week period of MCD diet was determined as the optimal time point to perform PHx, as at this stage only 10% surgery-and/or therapy related mortality was observed. In contrast, 5 weeks of MCD diet results in a higher degree of mortality following surgery (data not shown).

In this translational experiment, we evaluated liver injury and (therapy-induced) liver regeneration via longitudinal measurement, in addition to end-stage invasive evaluation of liver morphology. Total body weight, liver weight and liver architecture all return to control values 9 weeks after cessation of MCD diet and PHx. However, it is clear from the provided long-term measurements of serum CHE and TG levels that these regenerated livers are functionally not yet at the same level as control rats. As it is believed that liver recovery after PHx is completed within one week in both healthy and steatotic livers, based on liver mass or transaminase levels [28,29], this study suggests that liver function is not completely recovered 9 weeks after surgery of steatotic livers, based on serum CHE and TG levels. While evaluation of biochemical parameters in experimental studies is usually performed invasively at selected time-points [14,30,31] and/or non-invasively by e.g. MRI [32], we here demonstrate the significant advantage of longitudinal non-invasive measurement of serum parameters. These data clearly warrant the need for multiple longitudinal and complementary read-out methodologies to determine liver function following injury and during regeneration.

For administered BM-MSC to display therapeutic benefit on liver regeneration, either direct or indirect, it is imperative for grafted cells to be targeted to the site of injury. Therefore, we first investigated the *in vivo* biodistribution of intravenously administered BM-MSC using *in vivo* BLI. Two hours post administration cell retention was observed in the lungs both by *in vivo* and *ex vivo* BLI. In order to avoid potential side effects of this administration route for BM-MSC, which might include asphyxia, apoptosis and inflammatory responses [25,33], this administration route was not further explored in subsequent therapeutic setting. In order to obtain BM-MSC retention in liver, both intraportal and intrasplenic administration routes have been described. As significant retention of BM-MSC in spleen is observed after intrasplenic injection [34,35] and accurate information regarding the long-term effect of BM-MSC in spleen is missing, we decided to administer BM-MSC intraportally. Independent of the preceding control or MCD diet, one week after PHx, administered BM-MSC could be detected by *in vivo* BLI within the liver at 2 hours post-administration. Of note, a PHx (or the intrinsic liver remodelling effects thereafter) seem to be necessary for BM-MSC retention in the liver, as only limited cell retention was observed following intraportal administration of BM-MSC in healthy livers. Currently, we do not know the exact mechanism behind BM-MSC retention in the liver following intraportal administration at one week after PHx, as it can be explained by active homing and/or passive retention. Especially for the latter, we cannot exclude that grafted BM-MSC become entrapped into the microcirculation of the liver after PHx, as the latter is reduced with 70% without alteration in blood flow [29,36]. Following this reasoning, we have to note that in our experimental set-up we were unable to administer BM-MSC directly after PHx via the portal vein, as this procedure was associated with a high mortality rate (75%). Minimal cell graft related direct mortality (10%) was observed after IP cell administration at one week after PHx. Therefore, further research need to be undertaken in order to claim the safety of intraportal BM-MSC administration after PHx in clinical applications.

Nevertheless, as BM-MSC could be targeted to the liver following IP administration, in the final part of this study we then investigated the long-term regenerative potential of BM-MSC administration following MCD diet and PHx. In this experimental set-up we could not attribute a clinical benefit to endogenous regenerative processes. Despite the fact that MSC are frequently suggested to exert clinical benefit on liver regeneration [14–17], the lack of therapeutic benefit in this study can be explained by several reasons. First, it cannot be excluded that potential therapeutic benefit of BM-MSC grafting is limited to selected types of liver injury (metabolic disorders versus acute liver diseases/injury) or the source/subtype of BM-MSC [37]. Secondly, some studies performed MSC differentiation into hepatocyte-like cells before transplantation, which was not the case in this study. Third, there is insufficient long-term survival of grafted BM-MSC to induce clinical benefit (see above), although no general reports exist regarding long-term survival of administered BM-MSC following liver or other injuries [38]. Fourth, the timing of cell administration could be determinative for functional outcome, as cell administration might need to be performed earlier after PHx [39]. Finally, one might also argue the choice of experimental model used to evaluate cell therapeutic approaches to increase recovery following PHx of steatotic liver. In the present study we chose to use the MCD diet model as this is currently the most extensively studied steatosis/steatohepatitis model [40]. In our hands, Lewis rats fed with MCD diet clearly develop steatosis, but not lobular inflammation and/or fibrosis. Moreover, in a preceding study we did not observe a difference in the development of steatosis between male and female rats (Boeykens et al., PLOS One in press). However, the absence of steatohepatitis may be caused by many factors, like variations in rat strain, vendor, age, gender, diet constitution, etc. [41–43]. Alternatively, a rodent high-fat diet model has been proposed to more closely mimic the development of human steatohepatitis [44], however a high-fat diet in rodents does not always induce steatosis or steatohepatitis [45].

In summary, despite not observing a clinical benefit in our study, our data do underscore the need for profound validation of cellular therapies for liver regeneration, as a significant negative side effect of intraportally administered BM-MSC following PHx of steatotic livers was observed. Given the observation that serum CHE of cell administered rats did not reach those of non-treated rats and dose of control rats, despite recovery to normal body weight, liver weight and NAS score, our data warrants certain caution to be considered for future clinical applications of intraportal administration of BM-MSC to promote liver regeneration or function.
